# Endoscopic detection of the gastric lesions of peripheral T-cell lymphoma

**DOI:** 10.3332/ecancer.2016.625

**Published:** 2016-03-03

**Authors:** Masaya Iwamuro, Kosuke Kimura, Eisei Kondo, Takahiro Nada, Eri Nakamura, Katsuyoshi Takata, Takehiro Tanaka, Fumio Otsuka, Tadashi Yoshino, Hiroyuki Okada

**Affiliations:** 1 Department of General Medicine, Okayama University Graduate School of Medicine, Dentistry, and Pharmaceutical Sciences, Okayama 700-8558, Japan; 2 Department of Gastroenterology and Hepatology, Okayama University Graduate School of Medicine, Dentistry, and Pharmaceutical Sciences, Okayama 700-8558, Japan; 3 Department of Pathology, Okayama University Graduate School of Medicine, Dentistry, and Pharmaceutical Sciences, Okayama 700-8558, Japan; 4 Department of Pathology, Okayama University Hospital, Okayama 700-8558, Japan; 5 Department of Endoscopy, Okayama University Hospital, Okayama 700-8558, Japan

**Keywords:** peripheral T-cell lymphoma, gastrointestinal endoscopes, gastric neoplasms, gastrointestinal lymphoma, microsurface structures

## Abstract

An 82-year-old Japanese man presented with a gastric involvement of peripheral T-cell lymphoma, not otherwise specified. Although gastrointestinal lesions were not detected on computed tomography, oesophagogastroduodenoscopy revealed a slight elevation of the gastric mucosa, with changes in mucosal colour and the presence of abnormal microvessels. This led to the prompt detection of gastric involvement in lymphoma. This case highlights the usefulness of detailed observation of the gastric mucosa for the endoscopic detection of gastric involvement of peripheral T-cell lymphoma.

## Introduction

The gastrointestinal tract is the most common site of extra-nodal involvement of lymphomas [1–3]. Among the gastrointestinal tract structures, the stomach is the most frequent site of involvement, followed by the small intestine and ileocecal area. Most cases of gastrointestinal lymphomas are of B-cell lineage. The gastrointestinal involvement of T-cell lymphomas and that of Hodgkin’s lymphoma is relatively infrequent.

Herein, we describe the findings of a patient with gastric involvement of peripheral T-cell lymphoma, not otherwise specified. On oesophagogastroduodenoscopy, the gastric lesion presented as a superficial lesion showing a slight elevation with changes of mucosal colour to off-white. Although the macroscopic features were not easily distinguishable from chronic gastritis, close examination and magnified observations showed elongation and distortion in the microvessels, which led to the prompt detection of lymphoma involvement.

## Case presentation

An 82-year-old Japanese man was referred to our hospital for further investigation of multiple lymphadenopathies in the submandibular, neck, axillae, and inguinal area, and oedema in both the legs, all of which had occurred one month before presentation at our facility. He had been experiencing loss of appetite for the past three months, but his weight was unchanged during the previous six months. The patient had been consuming cilostazol and ethyl icosapentate for the treatment of arteriosclerosis obliterans as well as sitagliptin for diabetes mellitus. He had no history of gastrointestinal or haematological disease. His body temperature was 37.4ºC. A physical examination revealed swelling of the submandibular, neck, axillae, and inguinal lymph nodes. Pitting oedema was observed in both his lower extremities. His liver and spleen were not palpable, and there were no eruptions on his skin. Laboratory examinations revealed significant eosinophilia (white blood cell count 13,460/mm^3^; eosinophil, 6865/mm^3^) and elevated levels of IgE (26,019 IU/mL, normal range: 0–170), soluble interleukin-2 receptor (3992 U/L, normal range: 122–496), lactate dehydrogenase (841 U/L, normal range: 120–240), alkaline phosphatase (426 U/L), C-reactive protein (1.07 mg/dL), and glycosylated haemoglobin A (9.4%). The result for antibodies against human T-lymphotropic virus type 1 (HTLV-1) was negative.

**Figure 1.**
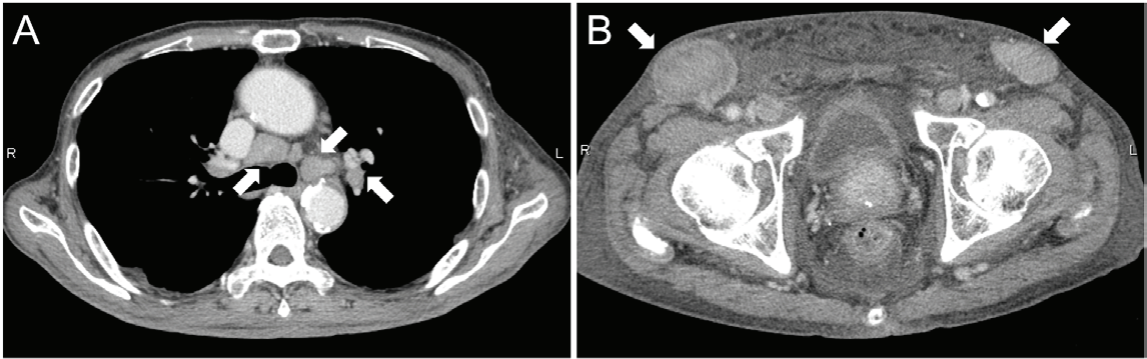
Computed tomography images. Multiple lymphadenopathies are observed in the hilar and mediastinal lymph nodes (A) and inguinal lymph nodes (B).

**Figure 2.**
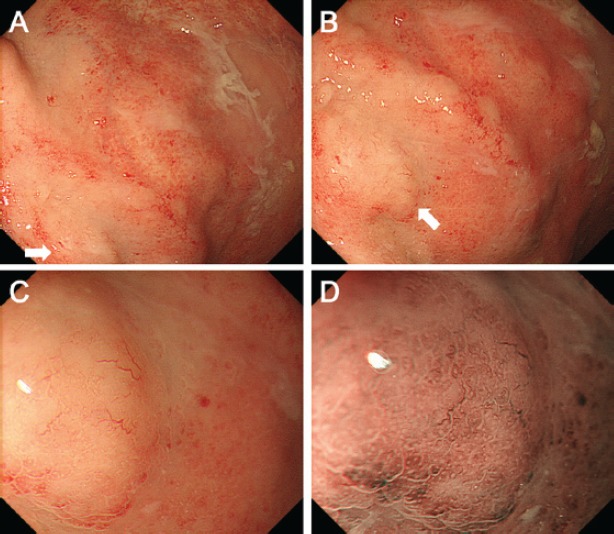
Oesophagogastroduodenoscopy images. A slight elevation of the mucosa and partial change in the mucosal colour to off-white are observed in the gastric body (A). Close examination (B) and magnified observation (C) showed elongated and distorted microvessels. Magnifying observation with narrow-band imaging revealed that the gastric pits were preserved (D).

**Figure 3.**
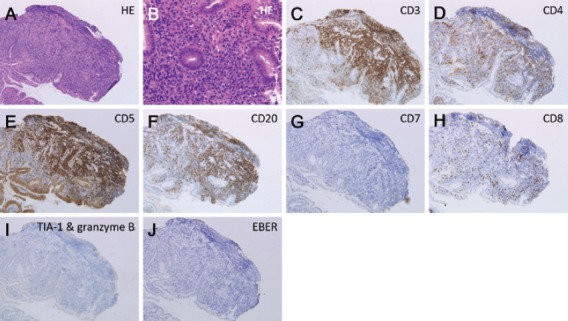
Pathological images. A biopsy examination taken from the gastric lesion revealed dense infiltration of atypical, medium-to-large lymphoid cells (A and B: Haematoxylin and eosin staining). Lymphoma cells were positive for CD3 (C), CD4 (D), and CD5 (E), while they were negative for CD20 (F), CD7 (G), CD8 (H), T-cell intracellular antigen-1, and granzyme B (I). The result of an in situ hybridisation for Epstein-Barr virus-encoded small RNA-1 was negative (J). A,C-J: ×10, B: ×40.

Contrast-enhanced computed tomography (CT) scanning showed lymphadenopathies of the hilar, mediastinal, and para-aortic areas, in addition to the submandibular, neck, axillae, and inguinal lymph node swelling (Figure 1). There were no significant findings in the gastrointestinal tract. A biopsy of the inguinal lymph node was performed. At the same time, oesophagogastroduodenoscopy was performed owing to the patient’s loss of appetite. The endoscopy revealed features of chronic gastritis, including mucosal atrophy in the lesser curvature of the gastric body and the entire antrum, as well as diffuse redness and sticky mucous in the greater curvature of the gastric body. A slight elevation of the mucosa and partial change in the mucosal colour to an off-white shade were also observed in the gastric body (Figure 2A). Close examination and magnified observation of the lesion revealed atypical microvessels (Figure 2B and C). Narrow-band imaging showed that the gastric pits were preserved (Figure 2D). A biopsy examination taken from the gastric lesion revealed dense infiltration of atypical, medium–to-large lymphoid cells (Figure 3A and B). These cells were positive for CD3, CD4, and CD5, but negative for CD20, CD7, CD8, T-cell intracellular antigen-1, and granzyme B (Figure 3C-I). The result of *in situ* hybridisation for Epstein-Barr virus-encoded small RNA-1 was negative (Figure 3J). Random biopsy sampling from the gastric antrum and the lesser curvature of the gastric body contained no lymphoma cells. In addition, pathological assessment of the inguinal lymph node showed medium-to-large lymphoid cells with immunophenotypic features identical to the gastric lesions. Consequently, a diagnosis of peripheral T-cell lymphoma with gastric involvement was made.

Chemotherapy with rituximab plus cyclophosphamide, doxorubicin, vincristine, and prednisone was initiated. After two courses of this treatment, partial reduction of lymphadenopathies were noted.

## Discussion

The morphology of gastric lesions of lymphomas is generally diverse, varying from a mass-forming tumour to diffuse infiltrating lesions to superficial mucosal changes [4]. Moreover, formation of multiple gastric lesions is frequently observed in lymphomas [5]. Gastric lymphomas can occur as a primary lesion or as a secondary involvement resulting from lymphoma progression of nodal origin. As described above, most cases presenting with gastric lymphoma are of B-cell lineage. Therefore articles describing gastric lesions of T-cell lymphoma, particularly those of peripheral T-cell lymphoma, have been limited. Reported macroscopic features of gastric lesions of peripheral T-cell lymphoma include a single or multiple ulcerative lesions [6–8], diffuse redness and erosive lesions [9], a mass lesion [5], and a submucosal tumour [10]. Consequently, gastric lesions of peripheral T-cell lymphoma seem to present with various morphologies similar to other lymphoma subtypes.

In the present patient, gastric involvement was shown as a slight elevation and partial change in the mucosal colour with atypical microvessels. Elongation and distortion of microvessels were clearly observed on magnified observations (Figure 2C and D). To our knowledge, magnified features of the gastric lesion of peripheral T-cell lymphoma have been reported only in a single article; Isomoto *et al* reported of a 75-year-old man with peripheral T-cell lymphoma who presented with a mass lesion in the gastric antrum [5]. Magnified observations revealed microvascular abnormalities as well as dilatation and destruction of gastric pits. Although the gastric pits were preserved in our case, both cases showed elongated and distorted microvessels. It has been reported that atypical microvessels are one of the characteristic microstructures of extra-nodal marginal zone lymphoma of the mucosa-associated lymphoid tissue (MALT) lymphoma, which is the most prevalent subtype among gastric lymphomas [11, 12]. Ono *et al* investigated the magnified features of ten cases with MALT lymphoma and reported that abnormal microvessels, in addition to the disappearance of gastric pits, were observed in all cases [11]. The forms of microvessels in MALT lymphoma cases were reportedly branched, spiral, and/or wavy. Therefore, we speculate that microvascular abnormality is a key feature to detect lymphoma lesions in the stomach. Although the diagnosis of peripheral T-cell lymphoma was made by using inguinal lymph node biopsy in our patient, precise endoscopic detection of gastrointestinal lymphoma lesions will likely be useful for pathological diagnosis in patients without peripheral lymphadenopathies.

Among T-cell lymphomas involving the gastrointestinal tract, enteropathy-associated T-cell lymphoma and adult T-cell lymphoma are far more common than peripheral T-cell lymphoma. Enteropathy-associated T-cell lymphoma is a neoplasm originating from intraepithelial T-lymphocytes in the intestinal tract, most frequently affecting the small intestine [13]. Strong association between this disease and coeliac disease has been suspected. Adult T-cell lymphoma occurs in patients infected by HTLV-1. This virus is endemic in the southeastern area of Japan, the Caribbean, and some areas of South and Central America and West Africa [14]. The presented patient was diagnosed with peripheral T-cell lymphoma involving the stomach rather than enteropathy-associated T-cell lymphoma or adult T-cell lymphoma because i) lymphadenopathy was predominant, but no tumourous lesions were detected in the intestines on CT scanning, ii) he had no history or symptoms relating to coeliac disease, and iii) the result on a test for HTLV-1 infection was negative. Our patient also presented with significant eosinophilia which is a well-known paraneoplastic feature of peripheral T-cell lymphoma [15, 16].

## Conclusion

We treated a patient with peripheral T-cell lymphoma involving the stomach. Oesophagogastroduodenoscopy showed a slight elevation of the gastric mucosa with changes in mucosal colour and the presence of abnormal microvessels. This case underscores the importance of detailed endoscopic observation, which will likely play a crucial role in the proper diagnosis of lymphoma lesions in the stomach.

## List of abbreviations

HTLV-1: human T-lymphotropic virus type 1
CT: Computed tomography
MALT lymphoma: extra-nodal marginal zone lymphoma of the mucosa-associated lymphoid tissue

## Conflicts of interest

The authors state that they have no conflict of interest.
